# Pericoronary Adipose Tissue Attenuation in Patients With Acute Coronary Syndrome Versus Stable Coronary Artery Disease

**DOI:** 10.1161/CIRCIMAGING.122.014672

**Published:** 2023-02-03

**Authors:** Jurrien H. Kuneman, Sophie E. van Rosendael, Pieter van der Bijl, Alexander R. van Rosendael, Pieter H. Kitslaar, Johan H.C. Reiber, J. Wouter Jukema, Martin B. Leon, Nina Ajmone Marsan, Juhani Knuuti, Jeroen J. Bax

**Affiliations:** Department of Cardiology, Leiden University Medical Center, The Netherlands (J.H.K., S.E.v.R., P.v.d.R., A.R.v.R., J.W.J., N.A.M., J.K., J.J.B.).; Division of Image Processing, Department of Radiology, Leiden University Medical Centre, The Netherlands (P.H.K.).; Medis Medical Imaging, Leiden, The Netherlands (P.H.K.).; Department of Radiology, Leiden University Medical Center, The Netherlands (J.H.C.R.).; Netherlands Heart Institute, Utrecht, The Netherlands (J.W.J.).; Department of Cardiology, Columbia University Irving Medical Center/New York-Presbyterian Hospital and Cardiovascular Research Foundation, NY (M.B.L.).; Turku PET Centre, Turku University Hospital and University of Turku, Finland (J.K.).; Heart Center, Turku University Hospital and University of Turku, Finland (J.J.B.).

**Keywords:** acute coronary syndrome, adipose tissue, angina, stable, atherosclerotic plaque, computed tomography angiography, coronary artery disease

## Abstract

Pericoronary adipose tissue (PCAT) attenuation has been associated with coronary inflammation and can be evaluated with coronary computed tomography angiography. The aims of this study were to compare the PCAT attenuation across precursors of culprit and nonculprit lesions of patients with acute coronary syndrome versus stable coronary artery disease (CAD).

**Methods:**

In this case-control study, patients with suspected CAD who underwent coronary computed tomography angiography were included. Patients who developed an acute coronary syndrome within 2 years after the coronary computed tomography angiography scan were identified, and patients with stable CAD (defined as any coronary plaque ≥30% luminal diameter stenosis) were 1:2 propensity score matched for age, sex, and cardiac risk factors. The mean PCAT attenuation was analyzed at lesion level and compared between precursors of culprit lesions, nonculprit lesions, and stable coronary plaques.

**Results:**

In total, 198 patients (age 62±10 years, 65% male) were selected, including 66 patients who developed an acute coronary syndrome and 132 propensity matched patients with stable CAD. Overall, 765 coronary lesions were analyzed (culprit lesion precursors: n=66; nonculprit lesion precursors: n=207; and stable lesions: n=492). Culprit lesion precursors had larger total plaque volume, fibro-fatty plaque volume, and low-attenuation plaque volume compared to nonculprit and stable lesions. The mean PCAT attenuation was significantly higher across culprit lesion precursors compared to nonculprit and stable lesions (−63.8±9.7 Hounsfield units versus −68.8±10.6 Hounsfield units versus −69.6±10.6 Hounsfield units, respectively; *P*<0.001), whereas the mean PCAT attenuation around nonculprit and stable lesions was not significantly different (*P*=0.99).

**Conclusions:**

The mean PCAT attenuation is significantly increased across culprit lesion precursors in patients with acute coronary syndrome, compared to nonculprit lesions of these patients and to lesions of patients with stable CAD, which may suggest a higher intensity of inflammation. PCAT attenuation on coronary computed tomography angiography may be a novel marker to identify high-risk plaques.

**Clinical Perspective**

Pericoronary adipose tissue attenuation on coronary computed tomography angiography has been associated with coronary artery inflammation, which is important in atherogenesis and plaque rupture. This study showed that the mean pericoronary adipose tissue attenuation across culprit lesion precursors of patients who developed an acute coronary syndrome is higher compared to nonculprit lesions in these patients, and compared to lesions of patients with stable coronary artery disease. Noninvasive detection of the pericoronary adipose tissue attenuation may improve identification of vulnerable plaques in patients at risk of developing an acute coronary syndrome. Prospective studies are necessary to evaluate the clinical implications of the coronary computed tomography angiography-derived pericoronary adipose tissue attenuation and future risk of acute coronary syndrome.

**See Editorial by Antonopoulos and Simantiris**

Atherosclerotic cardiovascular disease, particularly acute coronary syndrome (ACS), remains a major cause of mortality worldwide.^1^ Coronary atherosclerotic plaque rupture or erosion induces intraluminal thrombus formation and may cause an ACS.^2^ It has been shown that coronary artery inflammation is important in atherogenesis and atherosclerotic plaque rupture.^3,4^ In addition, inflammation of the coronary artery may change the characteristics of the surrounding adipose tissue, including smaller adipocyte size and lipid accumulation.^5,6^

Coronary computed tomography angiography (CCTA) is a valuable noninvasive test for diagnosing coronary artery disease (CAD).^7^ Moreover, it provides information on the location, stenosis severity, and composition of atherosclerotic plaques as well as risk stratification of patients with CAD.^8–10^ However, the ability to noninvasively detect vulnerable high-risk plaques that may cause an ACS is being explored. Recently, a landmark translational article using histological specimens demonstrated the association between the pericoronary adipose tissue (PCAT) attenuation, detected on CCTA, and coronary artery inflammation.^6^ Recent studies showed that higher PCAT attenuation, suggesting an increased level of inflammation, was associated with myocardial ischemia, plaque composition, and clinical outcomes.^11–15^ Whether there is an association between the PCAT attenuation and culprit lesions in ACS is currently being investigated. Accordingly, the aims of this study were to investigate the PCAT attenuation on CCTA of culprit and nonculprit lesion precursors of patients who developed an ACS, and lesions of patients with stable CAD.

## Methods

### Study Population

The data that support the findings of this study are available from the corresponding author upon reasonable request. Patients with suspected CAD who underwent clinically indicated CCTA between 2005 and 2015 at the Leiden University Medical Center (Leiden, The Netherlands) were included in this retrospective analysis. Patients who developed an atherothrombotic (type I) ACS within 2 years after the CCTA scan were identified. Patients with stable CAD, defined as the presence of any coronary plaque ≥30% luminal diameter stenosis, and without an ACS event within 2 years after the CCTA scan were 1:2 propensity score matched for age, sex, hypertension, hypercholesterolemia, diabetes, previous or current smoking, and family history of CAD. Patients with previous coronary revascularization (ie, coronary artery bypass grafting or percutaneous coronary intervention) were excluded, as well as patients with a CCTA scan performed at a tube voltage of 135 kV or insufficient image quality for quantitative analysis. ACS was classified as ST-segment elevation ACS, non-ST-segment elevation ACS, and unstable angina (Braunwald class III^16^), and was defined in accordance with the European Society of Cardiology and American College of Cardiology/American Heart Association guidelines.^17,18^ Culprit lesions in patients who developed an ACS were identified as reported by the primary operator in the invasive coronary angiography reports and were defined by angiographic findings suggestive of plaque rupture, as well as electrocardiographic and echocardiographic findings corresponding to regional wall motion abnormalities.^19^ Baseline demographical and clinical characteristics at the time of the CCTA scan were reported and compared between patients who developed an ACS and their matched controls with stable CAD. This study was performed according to the Declaration of Helsinki. The study protocol was approved by the local Ethics committee of the Leiden University Medical Center, Leiden, The Netherlands, who waived the need for written informed consent.

### CCTA Acquisition

CCTA was performed using either a 64-detector row (Aquilion 64, Toshiba Medical Systems, Otawara, Japan) or a 320-detector row computed tomography scanner (AquilionOne; Toshiba Medical Systems, Tochigi-ken, Japan). CCTA acquisition has been described in detail previously.^20,21^ In short, oral metoprolol was administered 1 hour before the scan in patients with a heart rate ≥65 beats per minute, unless contraindicated. In addition, sublingual nitroglycerin (0.4 mg/dose, 1–2 doses per patient) was administered directly before the CCTA examination. Iodinated contrast infusion (60–80 mL of 400 mg iodine/mL at 4–4.5 mL/s, Iomeron 400, Bracco, Milan, Italy) was followed by a saline flush. The gantry rotation time was 350 millisecond, tube current 500 (373–540) mA, and tube voltage 100 or 120 kV depending on patient size. Whenever feasible, prospectively triggered acquisition was applied to reduce radiation dose. Prior to the contrast injection, a nonenhanced electrocardiographic-triggered computed tomography scan was performed to assess the coronary artery calcium score and was reported in Agatston units.

### CCTA Analysis

Anatomical CCTA evaluation was performed according to the current guidelines.^22^ Coronary artery stenosis was defined any atherosclerotic plaque ≥30% luminal diameter stenosis and graded as nonobstructive (<50% diameter stenosis), moderate (50–70% diameter stenosis), severe (70–90% diameter stenosis), or subtotal/occluded (≥90% diameter stenosis). Obstructive CAD was defined as any coronary plaque ≥50% luminal diameter stenosis. Quantitative plaque analysis was performed using dedicated software (QAngio CT Research Edition version 3.2.0.13, Medis Medical Imaging Systems, Leiden, The Netherlands) by an experienced reader, independent of the anatomical CCTA evaluation. Quantitative plaque analysis has been described in detail previously.^23^ In brief, a 3-dimensional coronary tree and its side branches were extracted from the CCTA data set. All coronary vessels >1.5 mm diameter were evaluated and each vessel and segment were automatically labeled.^22^ Multiplanar reconstructions were created for each coronary artery. Subsequently, the lumen and vessel wall were automatically detected and manually adjusted if necessary. All atherosclerotic lesions were detected and quantitatively analyzed. For each lesion, the software provided quantitative data for stenosis location, stenosis severity, and plaque composition. In addition, plaque volume (PV, in cubic millimeter) and PV according to plaque composition were determined using predefined intensity thresholds in Hounsfield units (HU): low-attenuation plaque −30 to 75 HU, fibro-fatty plaque 76 to 130 HU, fibrous plaque 131 to 350 HU, and calcified plaque >350 HU.^20,24^ Plaque characteristics at a per-patient level were calculated by summing the PV of the different plaque components of each lesion. Plaque burden was calculated per slice as (plaque area/vessel area)×100. Subsequently, the mean plaque burden was calculated by the mean values of each slice within the lesion. Chronic total occlusions were identified and quantified using a dedicated algorithm.^23^

### PCAT Attenuation Analysis

**Figure 1.**
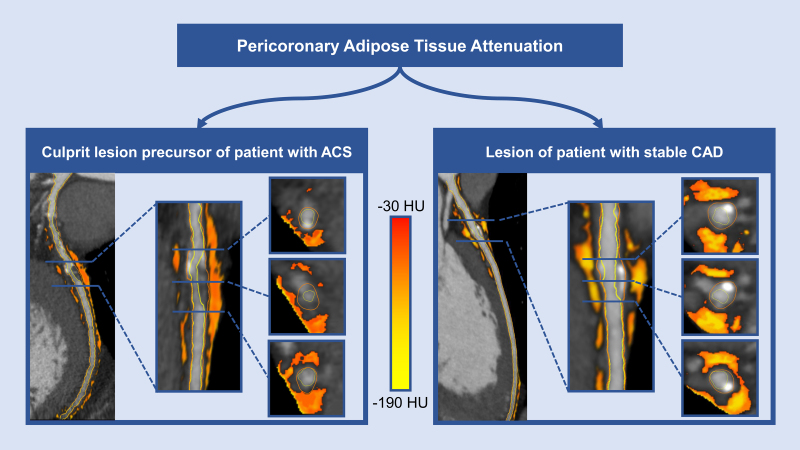
**Pericoronary adipose tissue (PCAT) attenuation on computed tomography angiography (CCTA).** Multiplanar reconstructions of CCTA scans showing mixed plaques in the proximal (**right**) and middle (**left**) left anterior descending coronary artery, with surrounding pericoronary adipose tissue (orange-yellow colored areas) across a precursor of a culprit lesion of a patient who developed an ACS (**left**) and across a lesion of a patient with stable CAD (**right**). ACS indicates acute coronary syndrome; CAD, coronary artery disease; and HU, Hounsfield units.

The PCAT was defined as tissue with an attenuation on CCTA between −190 and −30 HU and within a radial distance from the vessel wall equal to the vessel diameter.^6^ PCAT analysis was automatically performed by the software following quantitative plaque analysis (QAngio CT Research Edition version 3.2.0.13, Medis Medical Imaging Systems, Leiden, The Netherlands). For each lesion, the mean PCAT attenuation was evaluated across the entire lesion. Mean PCAT attenuation values were corrected for tube voltage and divided by a conversion factor of 1.11485 if the CCTA scan was performed at a tube voltage of 100 kV, as previously described.^6,11^ Quantitative plaque characteristics and the mean PCAT attenuation were compared between precursors of culprit lesions and nonculprit lesions of patients who developed an ACS and lesions of patients with stable CAD (Figure 1).

### Statistical Analysis

Continuous variables following a normal distribution are presented as mean±SD and were compared using the independent Student *t* test (for comparing patients who developed an ACS versus stable CAD) or the mixed model analysis of variance test (for comparing culprit lesion precursors versus nonculprit lesion precursors of patients who developed an ACS versus stable lesions). Nonnormally distributed continuous variables are presented as median with [interquartile range] and were compared using the Mann-Whitney *U* test or Kruskal Wallis test. Bonferroni post hoc analysis was performed to assess between-group differences in case of a significant difference in the overall 3 group comparison. Distribution of continuous variables was evaluated using histograms and Q-Q plots. Categorical variables are presented as absolute numbers and percentages and were compared using the χ^2^ test. Propensity scores were estimated by multivariable logistic regression analysis and included cardiovascular risk factors as age, sex, hypertension, hypercholesterolemia, diabetes, previous or current smoking, and family history of CAD. The propensity score was used to match patients who developed an ACS and those with stable CAD in a 1:2 ratio using nearest neighbor matching with a caliper of 0.010. PCAT attenuation analysis was repeated in 10 randomly selected patients by the same observer and by a second observer, blinded to the measurements of the first observer, to test the intra and interobserver agreement, respectively. The intra and interobserver agreements of the PCAT attenuation analysis were calculated using the intraclass correlation coefficient, with excellent agreement defined as an intraclass correlation coefficient >0.9. The intraclass correlation coefficient for the intra and interobserver agreement of the PCAT attenuation analysis were excellent: 0.988 (95% CI, 0.973–0.995) and 0.984 (95% CI, 0.964–0.993), respectively. For all tests, a 2-sided *P*-value <0.05 was considered significant. Statistical analyses were performed with SPSS version 25.0 (IBM SPSS Statistics, IBM Corporation, Armonk, New York, USA).

## Results

### Patient Characteristics

In total, 2886 patients with suspected CAD underwent a clinically indicated CCTA scan between 2005 and 2015. Of these, 198 patients (age 62±10 years, 65% male) were analyzed, including 66 patients who developed an ACS and 132 propensity matched patients with stable CAD (who did not experienced an event during follow-up). Of the 66 patients who developed an ACS, ST-segment elevation ACS was present in 6 patients (9%), non-ST-segment elevation ACS in 19 patients (29%), and unstable angina in 41 (62%). The baseline demographic and clinical characteristics (at the time of the CCTA scan) of the overall population and of patients who developed an ACS versus patients with stable CAD are summarized in Table 1. Patients who developed an ACS more frequently experienced typical chest pain compared to patients with stable CAD, whereas patients with stable CAD were more often asymptomatic or had dyspnea (70% versus 15%, 2% versus 14%, and 5% versus 23%, respectively; *P*<0.001). Moreover, patients who developed an ACS were more frequently using nitrates and antiplatelet therapy compared to the matched patients with stable CAD (12% versus 2% and 44% versus 24%, respectively; *P*=0.004 for both).

**Table 1.**
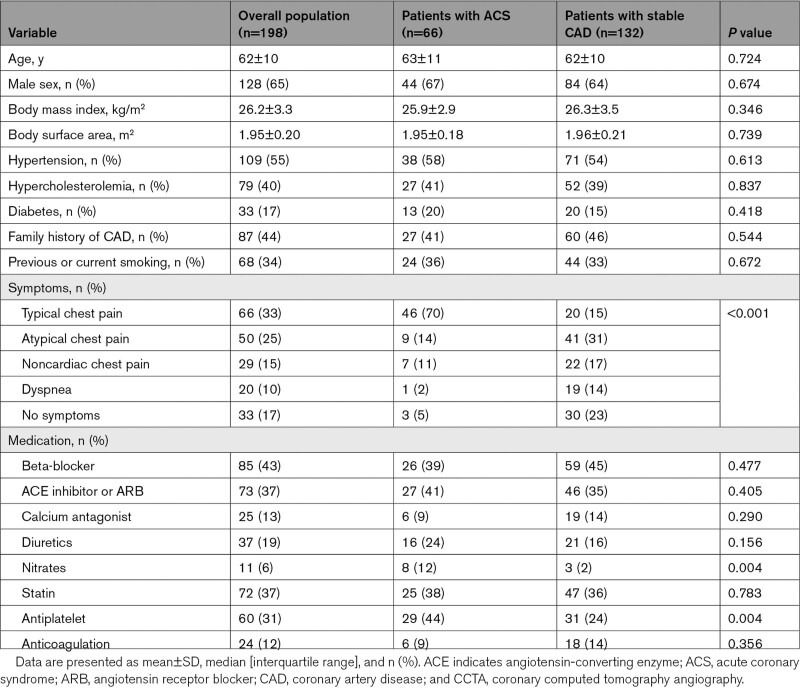
Baseline (at Time of CCTA Scan) Demographical and Clinical Characteristics

### Per-Patient CCTA Characteristics

**Figure 2.**
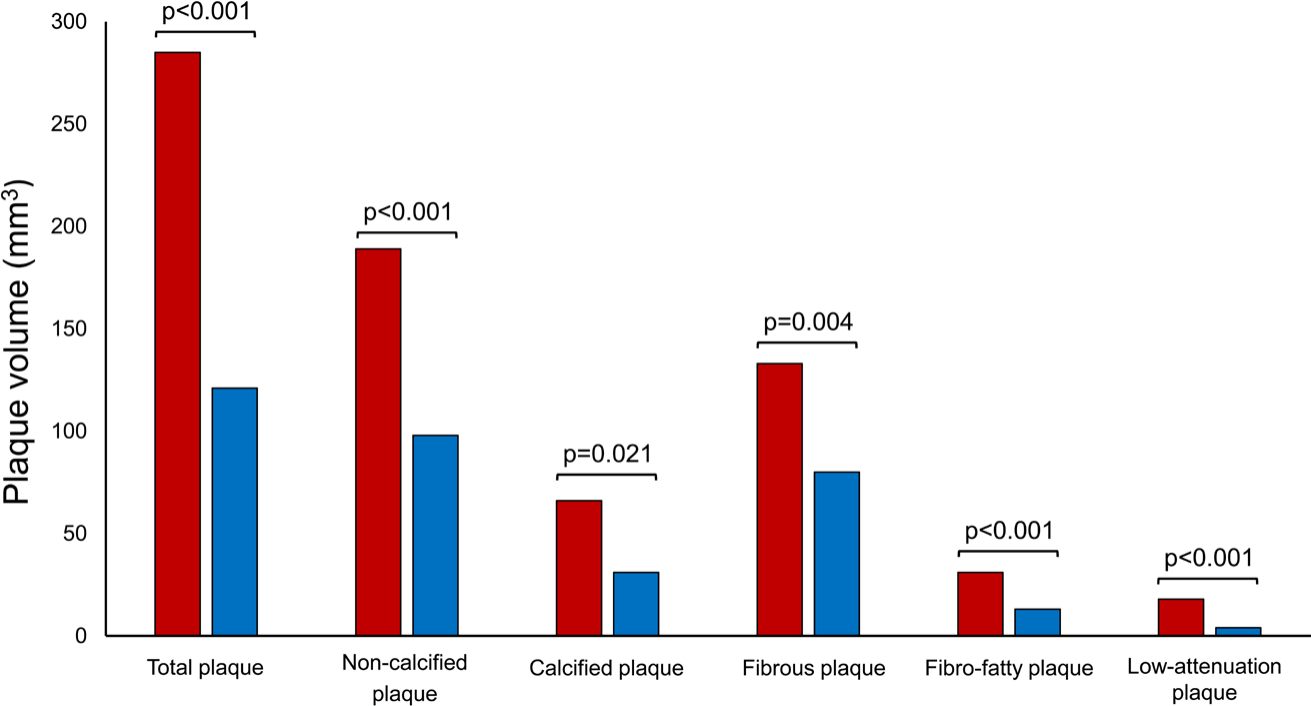
**Bar chart demonstrating the plaque volumes of the various plaque components.** Median plaque volumes of the total plaque volume and the plaque volumes of the various plaque components in patients who developed an ACS (red) and patients with stable CAD (blue). *P*-values represent group differences with the Mann-Whitney *U* test. Plaque composition intensity thresholds: calcified plaque >350 Hounsfield units (HU), noncalcified plaque −75 to 350 HU, fibrous plaque 131 to 350 HU, fibro-fatty plaque 76 to 130 HU, low-attenuation plaque −30 to 75 HU. ACS indicates acute coronary syndrome; and CAD, coronary artery disease.

The main CCTA characteristics of the overall population and of patients who developed an ACS versus those with stable CAD are presented in Table 2. Patients who developed an ACS had a higher coronary artery calcium score compared to patients with stable CAD (223 [72–714] versus 60 [7–330]; *P*=0.001) and more often had obstructive stenosis (79% versus 41%; *P*<0.001) on CCTA. Quantitative plaque analysis showed a larger total PV and PV of any plaque component in patients who developed an ACS compared to those with stable CAD (total PV: 285 [127–478] mm^3^ versus 121 [46–329] mm^3^, *P*<0.001; calcified PV: 66 [19–129] mm^3^ versus 31 [4–95] mm^3^, *P*=0.021; fibrous PV: 133 [55–240] mm^3^ versus 80 [29–177] mm^3^, *P*=0.004; fibro-fatty PV: 31 [15–57] mm^3^ versus 13 [5–32] mm^3^, *P*<0.001; and low-attenuation PV: 18 [7–31] mm^3^ versus 4 [1–14] mm^3^, *P*<0.001; Figure 2).

**Table 2.**
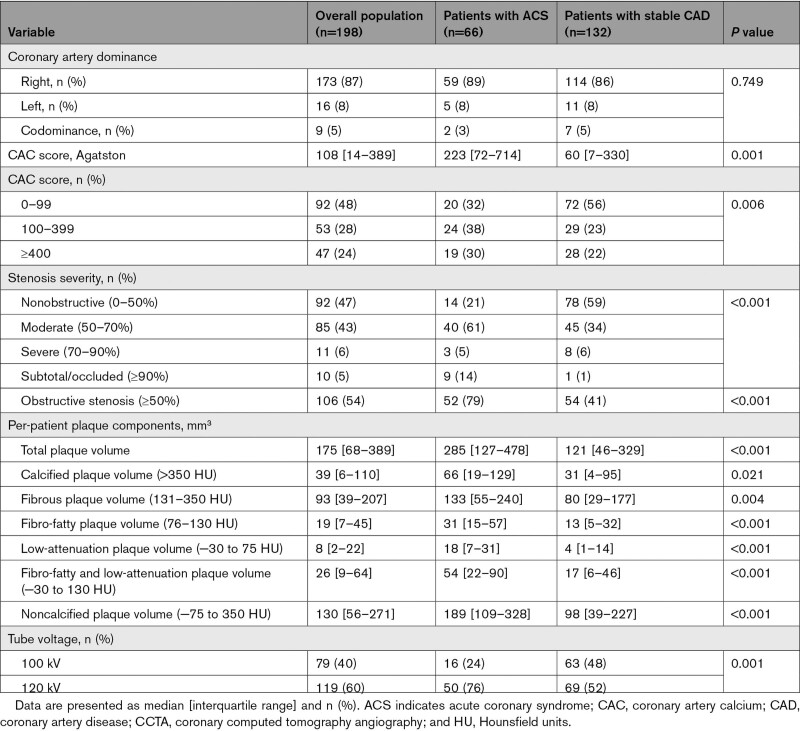
Per-Patient CCTA Results

### Per-Lesion Quantitative Plaque Analysis

Quantitative plaque characteristics of precursors of culprit lesions and nonculprit lesions of patients who developed an ACS and stable CAD are shown in Table 3. Culprit lesion precursors were more proximally located compared to nonculprit lesion precursors of patients who developed an ACS and lesions of patients with stable CAD (71% versus 37% versus 46%; *P*<0.001). The lesion locations overall and per vessel are shown in Table S1. Moreover, lesion length (12.6 [8.7–18.5] mm versus 9.5 [6.9–14.8] mm versus 8.4 [5.8–13.5] mm, respectively; *P*<0.001) and plaque burden (57.1±16.7% versus 50.0±11.% versus 46.6±11.2%, respectively; *P*<0.001) were greater in culprit lesion precursors. In addition, culprit lesion precursors had larger PV and PV of any plaque component except for calcified plaque compared to nonculprit lesions of patients who developed an ACS and those of patients with stable CAD (PV: 92 [37–134] mm^3^ versus 49 [28–94] mm^3^ versus 44 [26–84] mm^3^, *P*<0.001; fibrous PV: 52.8 [20.5–70.9] mm^3^ versus 27.5 [13.2–5.7] mm^3^ versus 25.1 [15.6–45.3] mm^3^, *P*=0.002; fibro-fatty PV: 8.6 [5.5–13.8] mm^3^ versus 6.5 [3.4–10.1] mm^3^ versus 4.2 [2.4–7.8] mm^3^, *P*<0.001; and low-attenuation PV: 3.8 [0.9–8.0] mm^3^ versus 2.4 [0.8–5.0] mm^3^ versus 1.4 [0.4–3.4] mm^3^, respectively; *P*<0.001). All plaque components were significantly different between groups on post hoc analysis.

**Table 3.**
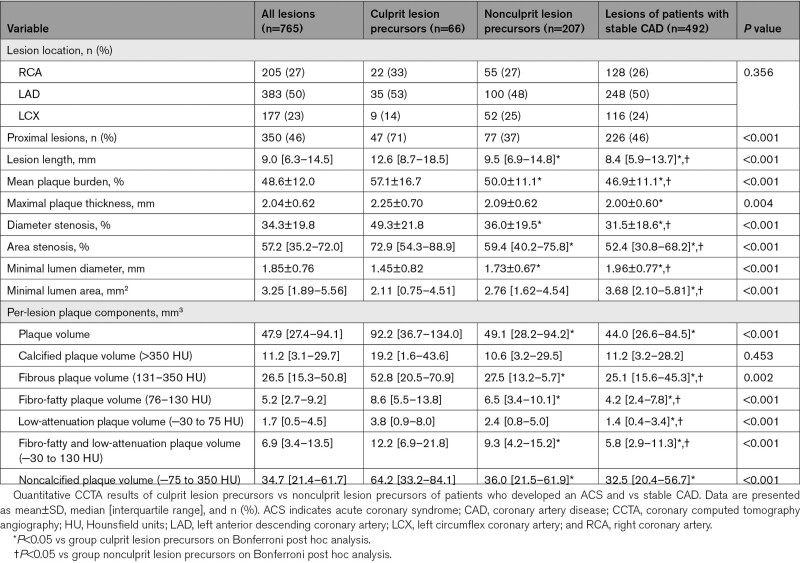
Per-Lesion Quantitative CCTA Results

### PCAT Attenuation

**Figure 3.**
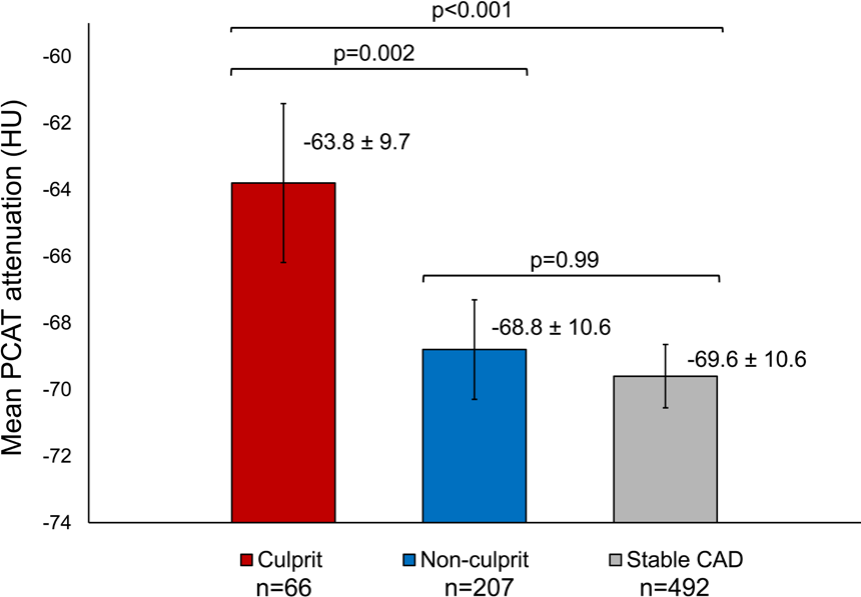
**Bar chart demonstrating the mean pericoronary adipose tissue (PCAT) attenuation among coronary lesions.** Mean PCAT attenuation across precursors of culprit lesions vs nonculprit lesions of patients who developed an ACS vs lesions of patients with stable CAD. Values are presented as mean±SD. Error bars indicate 95% CIs. *P*-values represent between-group differences with mixed model 1-way analysis of variance and Bonferroni post hoc analysis. ACS indicates acute coronary syndrome; CAD, coronary artery disease; and HU, Hounsfield units.

The mean PCAT attenuation was significantly different between precursors of culprit lesions and nonculprit lesions of patients who developed an ACS versus lesions of patients with stable CAD (−63.8±9.7 HU versus −68.8±10.6 HU versus −69.6±10.6 HU, respectively; *P*<0.001; Figure 3). Post hoc testing revealed that the mean PCAT attenuation was significantly higher in culprit lesion precursors versus nonculprit lesion precursors of patients who developed an ACS (mean difference: 5.2 [95% CI, 1.6–8.7] HU; *P*=0.002) and versus lesions of patients with stable CAD (mean difference, 5.9 [95% CI, 2.6–9.2] HU; *P*<0.001), whereas the mean PCAT attenuation was comparable between nonculprit lesions of patients who developed an ACS versus lesions of patients with stable CAD (mean difference, 0.8 [95% CI, −1.3 to 2.9] HU; *P*=0.99). Quantitative plaque characteristics and the mean PCAT attenuation of culprit lesion precursors versus nonculprit lesions of patients who developed an ACS and versus lesions of patients with stable CAD with the largest diameter stenosis showed similar results and are summarized in Table S2.

## Discussion

In this case-control study, the mean PCAT attenuation was evaluated in patients who developed an ACS versus matched controls with stable CAD. The mean PCAT attenuation was significantly increased across precursors of culprit lesions compared to nonculprit lesions of patients who developed an ACS and lesions of patients with stable CAD, which suggests a higher intensity of inflammation.

### Coronary Artery Inflammation

Coronary atherosclerosis is a chronic inflammatory condition. Immune and inflammatory cells are predominant in early atheroma formation and their cytokines are important in plaque progression.^3,4^ Moreover, inflammation may destabilize lesions and elicit atherosclerotic plaque rupture.^4^ Subsequently, prothrombotic plaque components (including phospholipids, tissue factor, and platelet-adhesive matrix molecules) are exposed to the lumen, causing intraplaque hemorrhage and intraluminal thrombus formation.^2,4^ This may lead to (partial) occlusion of the coronary artery precipitating myocardial ischemia and infarction.^2^ Coronary artery inflammation alters the morphologic and functional characteristics of the PCAT, leading to smaller adipocytes and lower lipid content, with a subsequently increased aqueous component.^6^ This results in higher PCAT attenuation on CCTA and can be analyzed noninvasively, as demonstrated recently in a biopsy-proven study by Antonopoulos et al.^6^

### PCAT Attenuation in ACS Versus Stable CAD

It is assumed that culprit lesions in ACS patients demonstrate increased inflammatory activity. The current study showed that culprit lesion precursors of patients who developed an ACS had higher PCAT attenuation, indicating a higher level of inflammation, compared to nonculprit lesions and lesions of patients with stable CAD. These results suggest that noninvasive evaluation of the PCAT attenuation on CCTA may potentially improve identification of vulnerable plaques in patients at risk of developing an ACS.

In a case-control study by Goeller et al,^25^ the PCAT attenuation was evaluated in 16 patients with an ACS versus 19 matched controls with stable CAD. The PCAT attenuation across culprit lesions of ACS patients was significantly higher compared to nonculprit lesions of these patients and compared to lesions of controls; similar to our results. In addition, a significantly higher low-attenuation PV was noted in culprit versus nonculprit lesions and versus lesions of patients with stable CAD, whereas calcified plaque burden was comparable between groups. These differences in plaque characteristics between culprit, nonculprit, and stable lesions are consistent with our findings.

Other studies, which investigated patients with (or who experienced) an ACS, measured the PCAT attenuation of the proximal coronary vessel segments, instead of a per-lesion approach as used in the present study. In a post hoc analysis of the Scottish Computed Tomography of the Heart trial, the PCAT attenuation of the proximal coronary vessel segments was evaluated among 1697 patients and with a median follow-up of 4.7 years.^26^ The PCAT attenuation of the right coronary artery was higher in patients who experienced an ACS, but this difference was not observed around the proximal parts of the left anterior descending and left circumflex artery. Moreover, the PCAT attenuation of the right coronary artery and low-attenuation plaque burden were independent predictors of myocardial infarction. In addition, total plaque burden and plaque burden of the various plaque components were higher in patients who had an event compared to those without, in agreement with the results obtained in the present study. Similarly, a case-control study by Lin et al^27^ prospectively recruited 60 patients who presented with an ACS and underwent CCTA within 48 hours of admission, and matched these groups to patients with stable CAD. Higher PCAT attenuation of the right coronary artery was noted in ACS patients compared to patients with stable CAD and those without CAD, independent of cardiovascular risk factors. The results suggest a higher intensity of coronary artery inflammation in ACS patients.

### Limitations

Several limitations should be acknowledged. First, this is a retrospective analysis with inherent limitations to the study design. Second, in this study, the PCAT attenuation was evaluated but not the PCAT volume. In addition, the PCAT attenuation was analyzed at a per-lesion level, whereas other studies reported the PCAT attenuation of proximal vessel segments. The optimal method for PCAT attenuation analysis has yet to be determined. Fourth, PCAT attenuation may be sex- and vessel-specific.^26^ However, the study population was propensity score matched for age, sex, and cardiovascular risk factors. In addition, the distribution of the lesion locations across the 3 main coronary vessels in this study was comparable between groups.

Last, this study showed a difference in PCAT attenuation between precursors of culprit lesions of patients who developed an ACS and nonculprit lesions and lesions of patients with stable CAD. Prospective studies are necessary to evaluate the clinical implications of the CCTA-derived PCAT attenuation values versus their future risk of ACS. Furthermore, the differences between PCAT attenuation values in the different clinical presentations (precursors of culprit lesions of patients who developed an ACS and nonculprit lesions and lesions of patients with stable CAD) on a lesion level are rather small. The current analysis permits differentiation between the 3 groups (precursors of culprit lesions of patients who developed an ACS and nonculprit lesions and lesions of patients with stable CAD), but whether these differences will allow prospective differentiation on an individual patient level needs prospective testing in future studies.

### Conclusions

The PCAT attenuation on CCTA, reflecting coronary artery inflammation, is significantly increased across culprit lesion precursors of patients who developed an ACS compared to nonculprit lesions of these same patients, as well as compared to lesions of patients with stable CAD. Noninvasive detection of the PCAT attenuation may potentially improve identification of vulnerable plaques in patients at risk of developing an ACS.

## Article Information

### Acknowledgments

The authors thank Mrs C.M. Cobbaert, EuSpLM, PhD (Department of Clinical Chemistry and Laboratory Medicine, Leiden University Medical Center, Leiden, The Netherlands) for her expertise and contribution to the methodology.

### Sources of Funding

None.

### Disclosures

The Department of Cardiology, Leiden University Medical Center, Leiden, The Netherlands has received unrestricted research grants from Bayer, Abbott Vascular, Medtronic, Biotronik, Boston Scientific, GE Healthcare, and Edwards Lifesciences. Dr Bax received speaker fees from Abbot Vascular. Dr Marsan received speaker fees from Abbott Vascular and GE Healthcare. Dr Knuuti received consultancy fees from GE Healthcare and AstraZeneca and speaker fees from GE Healthcare, Bayer, Lundbeck, Boehringer-Ingelheim, Pfizer, and Merck, outside of the submitted work. Dr Reiber is the CSO of Medis Medical Imaging Systems. Dr Leon has received institutional research support from Edwards Lifesciences, Medtronic, Boston Scientific, and Abbott; and consulting/advisory board participation for Medtronic, Boston Scientific, Gore, Meril Lifescience, and Abbott. Dr Jukema received research grants from the Netherlands Heart Foundation, the Interuniversity Cardiology Institute of the Netherlands, and the European Commission Seventh Framework Programme, and research support from Amgen, Astellas, AstraZeneca, Daiichi-Sankyo, Lilly, Merck-Schering-Plough, Pfizer, Roche, and Sanofi. The other authors report no conflicts.

### Supplemental Material

Tables S1–S2

## Supplementary Material


