# Supplementary material for: Pericoronary Adipose Tissue Attenuation in Patients With Acute Coronary Syndrome Versus Stable Coronary Artery Disease
Source: Circ Cardiovasc Imaging. 2023 Feb 3;16(2):e014672. doi: 10.1161/CIRCIMAGING.122.014672 (PMC9946175; doi:10.1161/CIRCIMAGING.122.014672)
Supplement: Supplementary file 1 [file hci-16-e014672-s001.docx]

**SUPPLEMENTAL MATERIAL**

Table S1 p. 2

Table S2 p. 5

**Table S1.** Distribution of lesion locations per vessel and overall according to culprit lesions precursors, non-culprit lesion precursors, and lesions of patients with stable CAD.

| **Variable** | **All lesions (n=765)** | **Culprit Lesion Precursors**  **(n=66)** | **Non-culprit Lesion Precursors**  **(n=207)** | **Lesions of Patients with Stable CAD (n=492)** | **p-value** |
| --- | --- | --- | --- | --- | --- |
| Lesion location per vessel, n (%) |  |  |  |  |  |
| RCA |  |  |  |  |  |
| Proximal | 88 (42.9) | 14 (63.6) | 16 (29.1) | 58 (45.3) | 0.046 |
| Mid | 66 (32.2) | 6 (27.3) | 20 (36.4) | 40 (31.3) |  |
| Distal | 51 (24.9) | 2 (9.1) | 19 (34.5) | 30 (23.4) |  |
| LAD |  |  |  |  |  |
| Proximal | 185 (48.4) | 28 (80.0) | 37 (37.0) | 120 (48.6) | <0.001 |
| Mid | 149 (39.0) | 7 (20.0) | 48 (48.0) | 94 (38.1) |  |
| Distal | 48 (12.6) | 0 | 15 (15.0) | 33 (13.4) |  |
| LCX |  |  |  |  |  |
| Proximal | 117 (65.7) | 7 (77.8) | 36 (69.2) | 74 (63.2) | 0.477 |
| Mid | 52 (29.2) | 1 (11.1) | 15 (28.8) | 36 (30.8) |  |
| Distal | 9 (5.1) | 1 (11.1) | 1 (1.9) | 7 (6.0) |  |
|  |  |  |  |  |  |
| Lesion location overall, n (%) |  |  |  |  |  |
| RCA, proximal | 88 (11.5) | 14 (21.2) | 16 (7.7) | 58 (11.8) | 0.001 |
| RCA, mid | 66 (8.6) | 6 (9.1) | 20 (9.7) | 40 (8.1) |  |
| RCA, distal | 51 (6.7) | 2 (3.0) | 19 (9.2) | 30 (6.1) |  |
| LAD, proximal | 185 (24.2) | 28 (42.4) | 37 (17.9) | 120 (24.4) |  |
| LAD, mid | 149 (19.5) | 7 (10.6) | 48 (23.2) | 94 (19.1) |  |
| LAD, distal | 48 (6.3) | 0 | 15 (7.2) | 33 (6.7 |  |
| LCX, proximal | 117 (15.3) | 7 (10.6) | 36 (17.4) | 74 (15.0) |  |
| LCX, mid | 52 (6.8) | 1 (1.5) | 15 (7.2) | 36 (7.3) |  |
| LCX, distal | 9 (1.2) | 1 (1.5) | 1 (0.5 | 7 (1.4) |  |

Data are presented as mean ± SD, median [interquartile range], and n (%). CAD = coronary artery disease, LAD = left anterior descending coronary artery, LCX = left circumflex coronary artery, RCA = right coronary artery.

**Table S2**. **Per-lesion quantitative plaque characteristics with largest diameter stenosis.**
Quantitative plaque characteristics of precursors of culprit lesions versus the non-culprit lesions with largest luminal diameter stenosis of patients who developed an ACS versus lesions with largest luminal diameter stenosis of patients with stable CAD.

| **Variable** | **Culprit Lesion Precursors**  **(n=66)** | **Non-culprit Lesion Precursors**  **(n=59)** | **Lesions of Patients with Stable CAD (n=132)** | **p-value** |
| --- | --- | --- | --- | --- |
| Lesion location, n (%) |  |  |  |  |
| RCA | 22 (33) | 13 (22) | 30 (23) | 0.391 |
| LAD | 35 (53) | 35 (59) | 73 (55) |  |
| LCX | 9 (14) | 11 (19) | 29 (22) |  |
| Proximal lesions, n (%) | 47 (71) | 23 (39) | 73 (55) | 0.001 |
| Lesion length, mm | 12.6 [8.7-18.5] | 9.4 [7.2-18.0] | 8.1 [5.8-13.1]* | <0.001 |
| Mean plaque burden, % | 57.1 ± 16.7 | 53.9 ± 11.7 | 47.8 ± 11.6* | <0.001 |
| Maximal plaque thickness, mm | 2.25 ± 0.70 | 2.07 ± 0.62 | 1.86 ± 0.64* | <0.001 |
| Diameter stenosis, % | 49.3 ± 21.8 | 48.2 ± 21.3 | 43.4 ± 18.2 | 0.098 |
| Area stenosis, % | 72.9 [54.3-88.9] | 74.7 [58.5-87.4] | 66.8 [54.2-80.1] | 0.090 |
| Minimal lumen diameter, mm | 1.45 ± 0.82 | 1.43 ± 0.65 | 1.69 ± 0.68† | 0.018 |
| Minimal lumen area, mm^2^ | 2.11 [0.75-4.51] | 2.20 [0.88-3.40] | 2.88 [1.63-4.59]† | 0.018 |
| Per-lesion plaque components, mm^3^ |  |  |  |  |
| Plaque volume | 92.2 [36.7-134.0] | 48.8 [27.4-113.6]* | 38.2 [22.8-76.8]* | <0.001 |
| Calcified plaque volume (>350 HU) | 19.2 [1.6-43.6] | 6.6 [1.7-29.8] | 7.6 [0.6-23.8]* | 0.048 |
| Fibrous plaque volume (131-350 HU) | 52.8 [20.5-70.9] | 28.5 [13.4-55.1] | 23.7 [13.3-42.8]* | 0.001 |
| Fibro-fatty plaque volume (76-130 HU) | 8.6 [5.5-13.8] | 7.6 [3.6-12.5] | 3.8 [2.2-7.7]*† | <0.001 |
| Low-attenuation plaque volume (-30 to 75 HU) | 3.8 [0.9-8.0] | 2.5 [1.0-7.0] | 1.2 [0.3-4.1]*† | <0.001 |
| Fibro-fatty and low- attenuation plaque volume (-30-130 HU) | 12.2 [6.9-21.8] | 10.7 [5.8-17.4] | 5.1 [2.8-12.5]*† | <0.001 |
| Non-calcified plaque volume (-75-350 HU) | 64.2 [33.2-84.1] | 41.5 [21.3-81.7]* | 29.1 [20.2-54.7]* | <0.001 |
| Mean PCAT attenuation, HU | -63.7 ± 9.7 | -69.4 ± 10.4* | -67.8 ± 10.3* | 0.005 |

Data are presented as mean ± SD, median [interquartile range], and n (%). ACS = acute coronary syndrome, CAD = coronary artery disease, HU = Hounsfield units, LAD = left anterior descending coronary artery, LCX = left circumflex coronary artery, PCAT = pericoronary adipose tissue, RCA = right coronary artery. * p<0.05 versus group ‘Culprit Lesion Precursors’ on Bonferroni’s post hoc analysis. † p<0.05 versus group ‘Non-culprit Lesion Precursors’ on Bonferroni’s post hoc analysis.
